# Elevated Glucose and Interleukin-1*β* Differentially Affect Retinal Microglial Cell Proliferation

**DOI:** 10.1155/2017/4316316

**Published:** 2017-05-15

**Authors:** Filipa I. Baptista, Célia A. Aveleira, Áurea F. Castilho, António F. Ambrósio

**Affiliations:** ^1^Institute for Biomedical Imaging and Life Sciences (IBILI), Faculty of Medicine, University of Coimbra, Coimbra, Portugal; ^2^Center for Neuroscience and Cell Biology (CNC), University of Coimbra, Coimbra, Portugal; ^3^AIBILI, Coimbra, Portugal

## Abstract

Diabetic retinopathy is considered a neurovascular disorder, hyperglycemia being considered the main risk factor for this pathology. Diabetic retinopathy also presents features of a low-grade chronic inflammatory disease, including increased levels of cytokines in the retina, such as interleukin-1 beta (IL-1*β*). However, how high glucose and IL-1*β* affect the different retinal cell types remains to be clarified. In retinal neural cell cultures, we found that IL-1*β* and IL-1RI are present in microglia, macroglia, and neurons. Exposure of retinal neural cell cultures to high glucose upregulated both mRNA and protein levels of IL-1*β*. High glucose decreased microglial and macroglial cell proliferation, whereas IL-1*β* increased their proliferation. Interestingly, under high glucose condition, although the number of microglial cells decreased, they showed a less ramified morphology, suggesting a more activated state, as supported by the upregulation of the levels of ED-1, a marker of microglia activation. In conclusion, IL-1*β* might play a key role in diabetic retinopathy, affecting microglial and macroglial cells and ultimately contributing to neural changes observed in diabetic patients. Particularly, since IL-1*β* has an important role in retinal microglia activation and proliferation under diabetes, limiting IL-1*β*-triggered inflammatory processes may provide a new therapeutic strategy to prevent the progression of diabetic retinopathy.

## 1. Introduction

Diabetic retinopathy is a leading cause of vision loss and blindness in the western countries and the most common complication of diabetes. Hyperglycemia is considered the primary pathogenic factor underlying diabetic retinopathy, being the breakdown of blood-retinal barrier (BRB), one of the first alterations clinically evident, and a hallmark of the disease [1].

In fact, early signs of neural dysfunction in the retina, namely, alterations in electroretinograms and loss of colour and contrast sensitivity, occur before the detection of microvascular changes in diabetic patients and animals [2–5]. Despite the progress in understanding the pathogenesis of diabetic retinopathy, the mechanisms underlying neural dysfunction are far from being completely understood.

Growing evidence indicates that diabetic retinopathy has features of a low-grade chronic inflammatory disease. Several genes involved in inflammatory processes are upregulated early in the diabetic rat retina [6, 7]. In the vitreous fluid of diabetic patients, the levels of interleukin-1 beta (IL-1*β*), IL-6, and tumor necrosis factor (TNF) are increased [8–10]. In addition, an increase in the production of cytokines, such as IL-1*β* and TNF, expression of adhesion molecules, leukocyte adhesion, and vascular permeability [11–13] have been observed in the retina of diabetic animals. Moreover, in the retinas of streptozotocin-induced diabetic rats, the levels of IL-1*β* are also increased [14–17], and this was correlated with an increase in BRB permeability [12, 14]. It has been shown that Müller glial cells isolated from diabetic rats acquire a reactive phenotype in response to diabetes, increasing the expression of inflammation-related genes [16]. In cultured retinal cells exposed to high glucose, an increase in [Ca^2+^]_i_ triggered by activation of purinergic receptors was observed in both retinal neurons and microglial cells [18]. This enhanced calcium response may also contribute to the increase in the release of inflammatory mediators and neurotransmitters in diabetic retinas. Early retinal microglia activation is a common response in diabetic retinopathy and is associated with progressive neurodegeneration in the retina. Activation of microglia leads to an increase in their proliferation and migration, phagocytosis, and release of several proinflammatory mediators [19]. The retina has been viewed as an immune privileged tissue; however, strong evidence supports a role for microglia activation and local inflammation in the pathogenesis of diabetic retinopathy [17, 20, 21].

IL-1*β* is a proinflammatory cytokine known to upregulate a plethora of several inflammatory mediators, including IL-1*β* itself, TNF, inducible nitric oxide synthase, and chemokines [22–25]. IL-1*β* elicits responses in cells only through the activation of IL-1 type I receptor (IL-1RI) although it can also bind to IL-1 type II receptor (IL-1RII), a decoy receptor. Although an increase in retinal IL-1*β* levels has been described in diabetic conditions and correlated with the pathogenesis of diabetic retinopathy, it is still unclear which retinal cells express IL-1*β* and IL-1RI.

In order to better understand how high glucose and IL-1*β* impact retinal cells, we evaluated whether high glucose regulates IL-1*β* expression and investigated which retinal cell types produce IL-1*β* and express its receptor in primary retinal neural cell cultures. Importantly, we also evaluated the cell-specific effects of high glucose and IL-1*β* per se in retinal neural cell cultures to clarify which cell types are mainly affected.

## 2. Experimental Procedure

### 2.1. Ethics Statement

Procedures involving animals were conducted in accordance with the guidelines of the European Community directive for the use of animals in laboratory (2010/63/EU), translated to the Portuguese law in 2013 (Decreto-lei 113/2013), and in accordance with the Association for Research in Vision and Ophthalmology (ARVO) guidelines for the use of animals in vision research. The experiments were approved by our Institutional Ethics Committee (Comissão de Ética da Faculdade de Medicina da Universidade de Coimbra) (approval ID: FMUC/07/12). Moreover, people working with animals have received appropriate education (Federation of Laboratory Animal Science Associations (FELASA) course) as required by the Portuguese authorities, and all efforts were made to minimize animal suffering. Decapitation with surgical scissors was the method used to perform euthanasia of the Wistar rat pups (postnatal days 3–5).

### 2.2. Primary Cultures of Rat Retinal Neural Cells

Primary rat retinal neural cell cultures were obtained from the retinas of 3–5-day-old Wistar rats, as previously described [26, 27]. After 2 days in culture, cells were incubated with 25 mM D-glucose (final concentration 30 mM) or 25 mM D-mannitol (plus 5 mM glucose already present in cell culture media), which was used as an osmotic control, and maintained for further 7 days in culture. The concentration of glucose in control conditions was 5 mM. Cells were also exposed to IL-1*β* (10 ng/ml) or lipopolysaccharide (LPS; positive control for inflammation; 1 *μ*g/ml) at day in vitro (DIV) 8 for 24 h.

### 2.3. Western Blot Analysis

Western blotting analysis of cellular lysates of retinal neural cell cultures was performed as previously described [27] with minor changes. Equal amounts of protein were separated by sodium dodecyl sulphate-polyacrylamide gel electrophoresis (SDS-PAGE), using 8%–12% gels. After electrophoretic transfer and blocking, the membranes were incubated with primary antibodies (listed in Table 1) overnight at 4°C. The secondary antibodies used were anti-mouse or anti-rabbit alkaline phosphatase-linked IgG secondary antibody (1 : 20,000; GE Healthcare, UK). Protein immunoreactive bands were visualized with the enhanced chemifluorescence substrate (ECF; GE Healthcare). Fluorescence was detected on an imaging system (Thyphoon FLA 9000, GE Healthcare), and the digital quantification of bands' immunoreactivity was performed using ImageQuant 5.0 software (Molecular Dynamics Inc., Sunnyvale. CA, USA). *β*-Actin was used as a protein loading control.

### 2.4. Immunocytochemistry

Immunocytohemistry was performed as previously described [27]. The primary antibodies used are listed in Table 1. The secondary antibodies used were Alexa Fluor 594-conjugated anti-mouse IgG (1 : 250) and Alexa Fluor 488-conjugated anti-rabbit IgG (1 : 250). The nuclei were stained with DAPI (1 : 5000). Cells were visualized in a laser scanning confocal microscope LSM 710 META (Zeiss, Germany).

### 2.5. Assessment of Cell Viability

Cell viability of retinal neural cell cultures was evaluated as described previously [28].

### 2.6. Terminal Transferase dUTP Nick End Labeling (TUNEL) Staining

Cells undergoing apoptosis were identified by TUNEL assay using the DeadEnd Fluorimetric TUNEL system (Promega Corporation, USA) as previously described [29]. Cells were also stained with DAPI to label the nuclei. The images were acquired with an inverted fluorescence microscope (DM IRE2, Leica Microsystems, UK). At least a minimum of 10 random fields in each coverslip were counted.

### 2.7. Quantitative Real-Time PCR

Isolation of total RNA from retinal cells, cDNA synthesis, and qPCR was performed as previously described [30]. The primers for the target rat genes (IL-1*β*, NM_031512) and the reference gene (rat HPRT, NM_012583) were predesigned and validated by QIAGEN (QuantiTect Primers, QIAGEN).

### 2.8. ELISA

At DIV 9, the conditioned medium of each well containing primary retinal neural cells exposed to different experimental conditions (control, high glucose, mannitol, and LPS) was removed and stored at −80°C until performing the ELISA assay. Rat IL-1*β* ELISA development kit (PeproTech, UK) was used to measure the levels of IL-1*β* in retinal neural cell culture medium. Each sample was assayed in duplicate using 100 *μ*l of culture medium per well.

### 2.9. Flow Cytometry

The analysis of cells undergoing apoptosis was performed using the annexin V-FITC assay kit (BD Biosciences), following the manufacturer's instructions and using PI staining. The stained cells were analyzed with a FACSCalibur (Becton Dickinson, USA) equipped with a 488 nm argon laser and a 635 nm red diode laser. The collected events per sample were 20,000. Flow cytometry data was analyzed with CellQuest software (Becton Dickinson) and plotted as a function of fluorescence intensity FL-1 (green) versus FL-3 (red) fluorescence. We used annexin V-FITC (emission 518 nm) versus PI (propidium iodide) (emission 617 nm) to identify viable cells (annexin V^−^PI^−^), early apoptotic cells (annexin V^+^PI^−^), necrotic cells (annexin V^−^PI^+^), and late apoptotic cells (annexin V^+^PI^+^).

### 2.10. Statistical Analysis

Results are presented as mean ± SEM. Statistical significance was determined by using Student's *t* test or ANOVA, followed by Dunnett's post hoc test. Differences were considered significant for *p* < 0.05.

## 3. Results

### 3.1. Rat Retinal Neurons and Glial and Microglial Cells Express IL-1*β* and IL-1RI

IL-1*β* is a proinflammatory cytokine that can be synthesized by several cell types, such as leukocytes, endothelial cells, neurons, and glial cells. Since the retina is composed by different cell types that potentially may produce IL-1*β*, we first analyzed whether retinal cells present in culture (neurons, macroglial and microglial cells) were able to synthesize IL-1*β* and identified those that can be directly affected by IL-1*β*, that is, cells that express IL-RI. The expression of IL-1*β* and the distribution of IL-1RI in primary rat retinal neural cell cultures were investigated by double-labeling immunocytochemistry. Specific cell markers were used to identify the different cell types present in the cell culture: TUJ-1 (neurons), GFAP (macroglial cells), and Iba-1 or CD11b (microglial cells).

**Figure 1:**
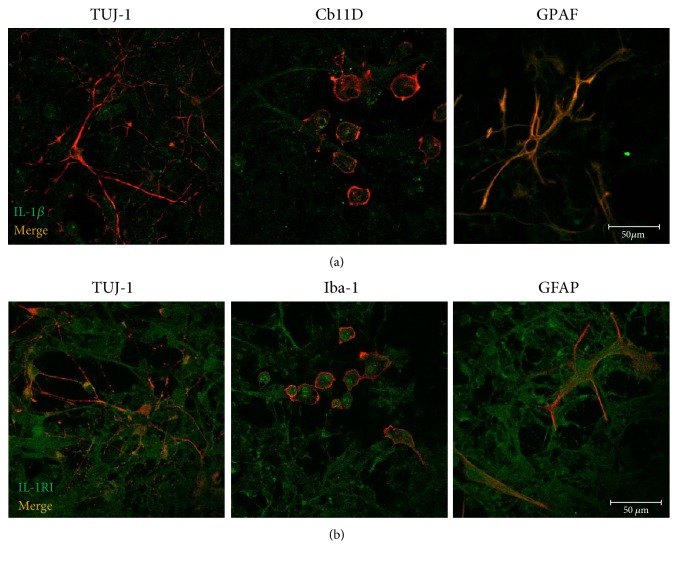
Rat retinal neurons and glial cells express IL-1*β* and IL-1RI. IL-1*β* (a) and IL-1RI (b) immunostaining (both in green) can be observed in rat retinal neurons (TUJ-1; red) and microglia (CD11b or iba-1; red) and macroglial cells (Müller cells and astrocytes; GFAP; red), indicating that retinal neural cells express IL-1*β* and can be responsive to it, since they express IL-1RI. Scale bar: 50 *μ*m.

As shown in Figure 1(a), IL-1*β* immunoreactivity is present in TUJ-1^+^, CD11b^+^, and GFAP^+^ cells. Similarly, IL-1R1^+^ cells were also immunoreactive to TUJ-1^+^, Iba-1^+^, and GFAP^+^ cells (Figure 1(b)). These observations indicate that retinal neural cells express IL-1*β* and can be responsive to it, since they also express IL-1RI.

### 3.2. High Glucose Increases IL-1*β* Expression in Rat Retinal Neural Cells

Since hyperglycemia is considered the main risk factor for diabetic retinopathy [27, 28] and the levels of IL-1*β* are increased in the retinas of diabetic rats [14–17], we evaluated the effect of elevated glucose on IL-1*β* expression in retinal neural cultures in order to evaluate if high glucose per se is capable of upregulating IL-1*β* expression.

**Figure 2:**
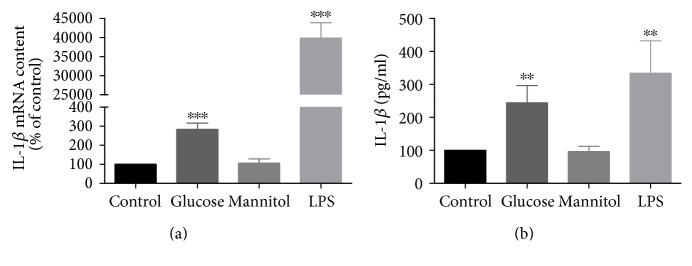
High glucose upregulates IL-1*β* expression in rat retinal cell cultures. Cells were exposed to 30 mM glucose or 24.5 mM mannitol (plus 5.5 mM glucose; osmotic control) for 7 days and IL-1*β* or LPS for 24 h. High glucose upregulates both IL-1*β* mRNA (a) and protein (b) levels. The results represent the mean ± SEM of at least 3 independent experiments and are presented as percentage of control. ^∗∗^*p* < 0.01 and ^∗∗∗^*p* < 0.001, significantly different from control, using Student's *t*-test.

Firstly, we evaluated IL-1*β* mRNA expression in these cultures by qPCR. A significant increase in IL-1*β* mRNA content was observed in high glucose-treated cells (283.2 ± 32.8% of the control) (Figure 2(a)). No changes were detected in cells exposed to mannitol, demonstrating that the effect of glucose was not due to the increase in osmolarity. In addition, a significant increase in IL-1*β* mRNA was observed in cells treated with LPS (increase to 39,868.7 ± 4043.4% of the control) (Figure 2(a)), a positive control for IL-1*β* gene expression upregulation.

To determine whether the increase in IL-1*β* mRNA expression was concomitant with an increase in the production of IL-1*β*, we evaluated its protein levels in the culture media of retinal neural cells by ELISA assay. A significant increase in IL-1*β* levels was detected upon high glucose treatment (increase to 244.52 ± 52.2% of the control) (Figure 2(b)). Again, no changes were detected in cells exposed to mannitol, demonstrating that the effect was not due to the increase in osmolarity. As expected, a significant increase in IL-1*β* levels was also detected in LPS-treated cells (333.8 ± 98.3% of the control) (Figure 2(b)).

### 3.3. IL-1*β* Does Not Induce Retinal Neural Cell Death

Previous studies from our laboratory [29, 30] demonstrated that exposure of cultured retinal cells for 7 days to high glucose decreases cell viability, which is concomitant with an increase in the number of apoptotic nuclei detected by TUNEL assay. Since IL-1*β* is an important mediator of the inflammatory responses, and is involved in a variety of cellular processes, including cell proliferation, differentiation, and apoptosis [31, 32], we evaluated whether exposure to IL-1*β* per se could increase apoptosis in retinal neural cells.

**Figure 3:**
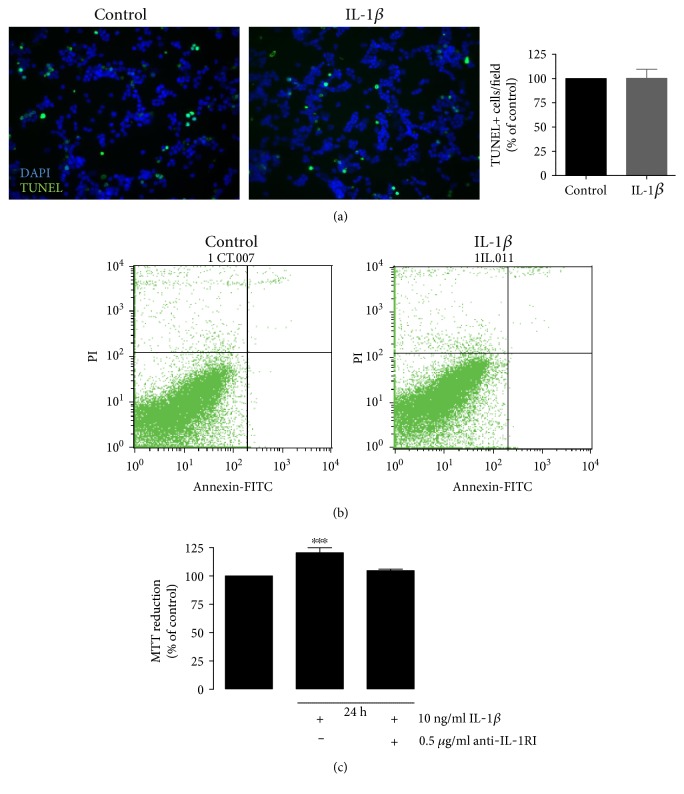
Exposure to IL-1*β* does not increase retinal neural cell death. Rat retinal neural cell cultures were exposed to IL-1*β* for 24 h, and cell death was evaluated by TUNEL assay. Representative images and quantification of the number of TUNEL^+^ cells upon IL-1*β* treatment are shown. Scale bar: 50 *μ*m (a). To further evaluate cell death, retinal cells were labeled with annexin V plus PI and were analyzed by flow cytometry (b). MTT was used as a cell viability assay (c). The results represent the mean ± SEM of, at least, 4 independent experiments. ^∗∗∗^*p* < 0.001, significantly different from control as determined by ANOVA followed by Dunnett's post hoc test.

Retinal cell cultures were exposed to IL-1*β* for 24 h, and retinal cell viability was evaluated by TUNEL assay and flow cytometry. IL-1*β* did not increase the number of TUNEL^+^ cells as compared to control conditions (Figure 3(a)). To confirm these results, we additionally performed flow cytometry with annexin V and PI, aiming to distinguish the features of apoptotic versus viable cells. A typical representative dot plot analysis of retinal cells after exposure to IL-1*β* is shown in Figure 3(b). Exposure to IL-1*β* for 24 h did not increase cell death in these cultures. Dot plot analysis showed that 94.8 ± 0.3% of cells exposed to IL-1*β* are viable, similar to control conditions (94.5 ± 0.5% of the cells were viable). Interestingly, the exposure of retinal neural cells to IL-1*β* for 24 h induced an increase in the reduction of MTT (120.5 ± 4.4% of the control; Figure 3(c)), which is frequently used as a viability or proliferative assay. Since IL-1*β* did not increase cell death, this observation suggests that IL-1*β* was enhancing cell proliferation. In order to verify whether the increase in the MTT reduction induced by IL-1*β* was mediated by the activation of IL-1RI, retinal cells were exposed to IL-1*β* together with an antibody against IL-1RI. The presence of the antibody prevented the increase in the MTT reduction induced by IL-1*β* (Figure 3(c)).

### 3.4. High Glucose and IL-1*β* Differently Impact on Retinal Microglial Cell Proliferation

**Figure 4:**
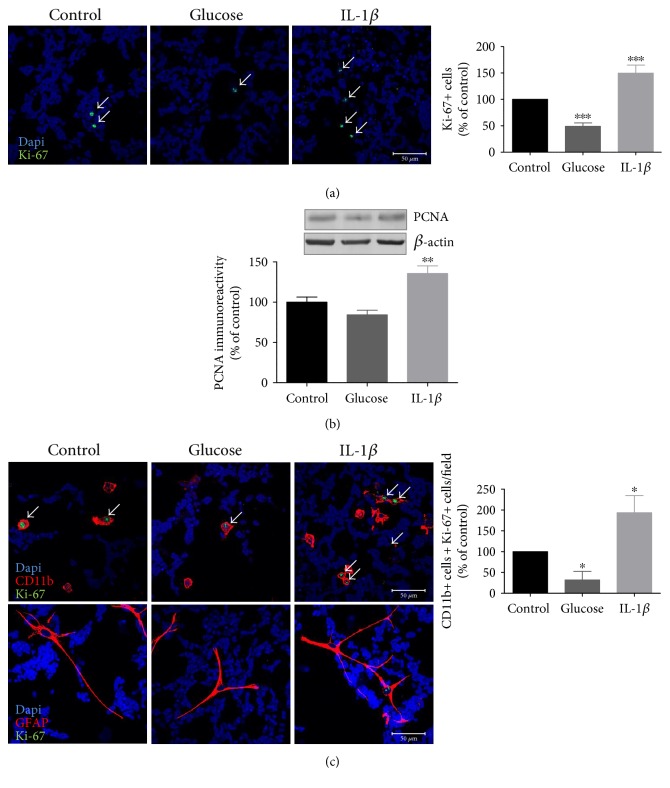
High glucose and IL-1*β* differently affect cell proliferation retinal neural cell cultures. Rat retinal neural cell cultures were exposed to high glucose for 7 days or IL-1*β* for 24 h and stained for Ki-67. Representative images and quantification of the number of Ki-67^+^ cells upon high glucose and IL-1*β* treatment are shown. Scale bar: 50 *μ*m (a). PCNA protein levels were evaluated by Western blotting. Representative Western blots are presented above the graphs, with the respective loading controls (*β*-actin) (b). To evaluate whether microglial and macroglial cells are proliferating, colocalization of CD11b^+^ or GFAP^+^ cells with Ki-67 was performed. Representative images and quantification of the number of CD11b^+^Ki-67^+^cells or GFAP^+^Ki-67^+^cells upon high glucose and IL-1*β* treatment are shown. Scale bar: 50 *μ*m (c). Data represent means ± SEM of, at least, 7 independent experiments. ^∗^*p* < 0.05, significantly different from control as determined by ANOVA followed by Dunnett's post hoc test.

Since we have detected an increase in the MTT reduction and no changes in the number of TUNEL^+^ cells in cultures exposed to IL-1*β*, we hypothesized that IL-1*β* could be affecting the proliferation of some cell types in these cultures. To evaluate this hypothesis, the proliferative capacity of retinal neural cells was assessed by Ki-67 or proliferating cell nuclear antigen (PCNA) immunoreactivity. Since elevated glucose increases the levels of IL-1*β* in retinal cultures, we also evaluated the effect of high glucose on retinal neural cell proliferation. As shown in Figure 4(a), a significant decrease in the number of Ki-67^+^ cells was observed in cells exposed to elevated glucose for 7 days (decrease to 49.07 ± 6.5% of control). No changes were observed in mannitol-treated cells (data not shown). Conversely, a significant increase in the number of Ki-67^+^ cells (increase to 149.8% ± 15.1% of control) was observed in cells incubated with IL-1*β* for 24 h (Figure 4(a)). Similar to what was observed with Ki-67 immunoreactivity, PCNA protein content decreased in high glucose-treated cells and increased in cells exposed to IL-1*β* (Figure 4(b)). These results suggest that prolonged exposure to high glucose and exposure to IL-1*β* induce opposite effects in cell proliferation.

To identify which cell types present in culture were proliferating, we performed double-labeling immunocytochemistry experiments using CD11b^+^ (microglial) or GFAP^+^ (macroglial) with Ki-67. Regarding microglial cells, there was a significant decrease in the number of Ki-67^+^CD11b^+^ cells (32.3% ± 20.5% of control) when retinal neural cells were exposed to elevated glucose, whereas in IL-1*β*-treated cells, a significant increase in the number of Ki-67^+^CD11b^+^ cells was observed (increase to 193.6% ± 41.1% of control) (Figure 4(c)). Concerning GFAP^+^Ki-67^+^ cells, very few or no positive cells were observed in control condition (average of 0.1 ± 0.1 Ki-67^+^ colocalizing with GFAP^+^ cells per field), while in cells exposed to high glucose, we did not detect any GFAP^+^Ki-67^+^ cells. When cells were exposed to IL-1*β*, the average number of Ki-67^+^ cells colocalizing with GFAP^+^ cells per field was of 0.5 ± 0.2, demonstrating an increase in macroglial cell proliferation (Figure 4(c)).

These results suggest that while high glucose decreases retinal glial cell proliferation, particularly in microglial cells, IL-1*β* increases both microglial and macroglial cell proliferation.

**Figure 5:**
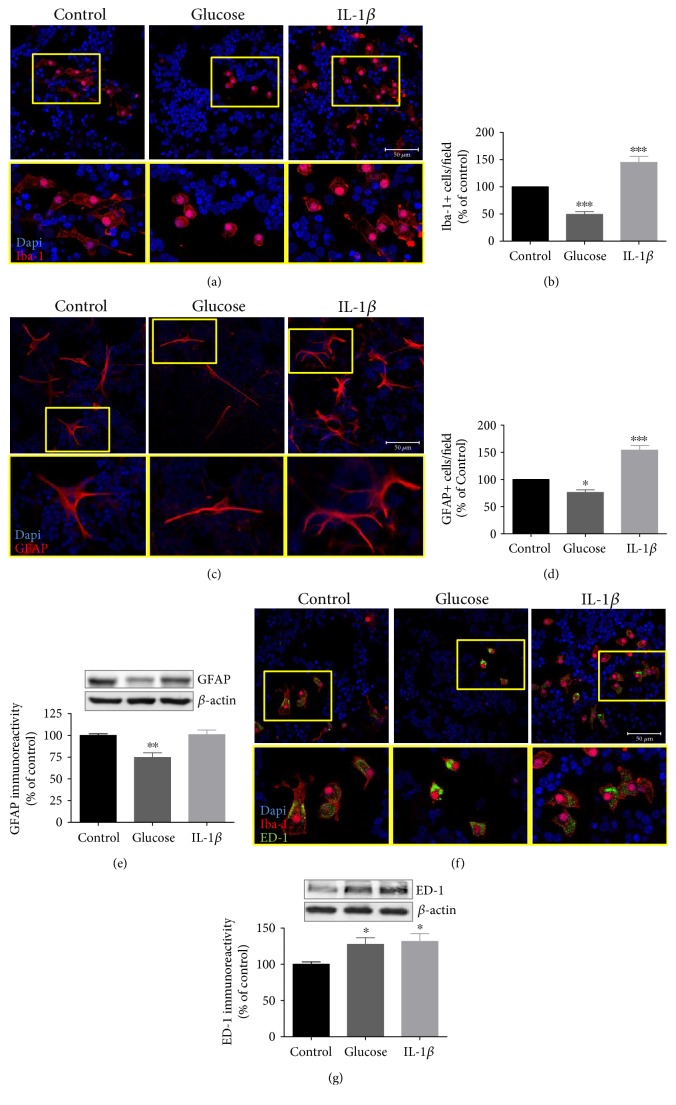
High glucose and IL-1*β* have opposite effects on retina microglial and macroglial cell proliferation. Rat retinal neural cell cultures were exposed to high glucose for 7 days or IL-1*β* for 24 h. Iba-1 immunoreactivity was assessed by immunocytochemistry (a, b). GFAP immunoreactivity was assessed by immunocytochemistry (c, d) or Western blotting (e). ED-1 immunoreactivity was assessed by immunocytochemistry (f) or Western blotting (g). Representative images (insets of higher magnifications images are shown below each panel) for Iba-1 (a), GFAP (c), or ED-1 (f) upon high glucose and IL-1*β* treatment are shown. Scale bar: 100 *μ*m for (c). Scale bar: 50 *μ*m for (a) and (f). Data represent means ± SEM of at least 4 independent experiments. ^∗^*p* < 0.05, ^∗∗∗^*p* < 0.001, significantly different from control as determined by ANOVA followed by Dunnett's post hoc test.

To support this observation, we next evaluated the effect of high glucose and IL-1*β* in the total number of both Iba-1^+^ and GFAP^+^ cells. Microglial cells are very sensitive to alterations in their microenvironment, as well as to changes in cell culture conditions. They are considered to be major producers of IL-1*β*, which in turn can further activate microglial cells. High glucose induced a significant decrease in the number of Iba-1^+^ cells (49.2 ± 5.1% of control). However, IL-1*β* induced a significant increase in the number of Iba-1^+^ cells (144.9 ± 10.9% of control), as shown in Figures 5(a) and 5(b). The number of GFAP^+^ cells was significantly decreased by high glucose (77.3 ± 1.6% of control), but strongly increased by IL-1*β* (153.9 ± 8.6% of control) (Figures 5(c) and 5(d)). Accordingly, GFAP protein content decreased in high glucose-treated cells (74.7 ± 5.4% of control). Nevertheless, no alterations on GFAP protein content were observed when cells were exposed to IL-1*β* (Figure 5(e)).

However, we noticed that not only the number but also the morphology of micro- and macroglial cells was altered. Microglial cells presented a round-shaped morphology with less processes, suggesting a more activated state. On the other hand, in IL-1*β*-treated cell cultures, microglia cells presented both round-shaped and ramified morphology (Figure 5(d)). Regarding macroglial cells, we observed that cells exposed to high glucose were less ramified (Figure 5(c)). To check whether microglial cells were activated, we evaluated ED-1 immunoreactivity (marker for activated microglia) by immunocytochemistry and Western blotting. Interestingly, a significant increase in ED-1 protein levels was detected in cells exposed to high glucose for 7 days (increase to 127.6 ± 9.0%) (Figure 5(g)). As expected, in IL-1*β*-treated cells, we also observed an increase in ED-1 protein levels (131.8 ± 10.9% of control) (Figures 5(f) and 5(g)).

## 4. Discussion

Hyperglycemia is considered the major risk factor for the development of diabetic retinopathy. Nevertheless, in the last decade, growing evidence has shown that diabetic retinopathy has features of a low-grade chronic inflammatory disease [21]. In fact, it has been shown that microglial and macroglial cell activation is an important feature of neuroinflammation present in the diabetic retina [17, 31–34]. Moreover, increased production of inflammatory mediators, such as IL-1*β* [21, 35], TNF-*α* [35], and NO [12, 21] has been reported in the retinas of diabetic animals.

The results presented in this work show that a prolonged exposure to high glucose upregulates IL-1*β* expression in retinal neural cell cultures. In the central nervous system, the main cellular source of IL-1*β* under stress conditions is activated microglia, with a consequent upregulation of the cytokine in macroglia as well [25]. However, a previous study demonstrated that exposure to high glucose, for 4 days, is not sufficient to upregulate IL-1*β* expression in microglial cells [36]. Nevertheless, this study was performed using brain-derived microglial cells and with less time of exposure to high glucose (4 days) than in our study (7 days exposure) in retinal cell cultures.

Our group has previously shown that a long-term exposure (7 days) to high glucose decreases cell viability in retinal neural cell cultures [30]. Exposure to high glucose increases [Ca^2+^]_i_ responses in neurons and microglial cells in retinal neural cell cultures [18]. These alterations may lead to neurotransmitter and proinflammatory mediator release contributing to retinal cell death. Moreover, we demonstrated that as early as 3 days of exposure to high glucose also induces a small, but significant, decrease in cell viability, and a constant decline in cell viability was found for longer incubation periods, reaching a minimum at the last time point studied, 7 days [37]. The increase in the number of apoptotic nuclei detected by TUNEL assay after 7 days of incubation with high glucose was concomitant with a decrease in cell viability assessed with the MTT assay [28, 37]. Using several markers to identify the type of cells undergoing apoptosis in retinal cultures exposed to high glucose, we showed that very few TUNEL^+^ cells were immunoreactive for neuronal (NeuN, TUJ-1), microglial (CD11b), or macroglial (GFAP, vimentin) cell markers. A high percentage of apoptotic cells in high glucose condition were ascribed to be photoreceptors and bipolar cells [37].

In the present study, exposure to IL-1*β* did not affect the number of apoptotic cells in retinal cell cultures. Interestingly, IL-1*β* increased the reduction of MTT. Despite being frequently used as a cell viability assay, it is also used as a proliferative assay. As high glucose decreases MTT reduction [28] and IL-1*β* increases it, these results lead us to hypothesize that exposure to high glucose and IL-1*β* have opposite effects on cell proliferation. In line with this, we further evaluated the effect of high glucose and IL-1*β* on cell proliferation by Ki-67 immunostaining. By cell counting, we detected a significant decrease in the number of CD11b^+^Ki-67^+^ cells and in the number of CD11b^+^ cells in retinal cultures exposed to high glucose. Given that it has not detected an increase in TUNEL^+^CD11b^+^ cells in these cultures exposed to high glucose [29], this observation suggests that the decrease was not due to the apoptosis of CD11b^+^ cells.

Since IL-1*β* increased the number of CD11b^+^ cells, and high glucose increased IL-1*β* production in retinal neural cell cultures, we were expecting an increase in CD11b^+^ cells in cultures exposed to high glucose. However, as mentioned above, high glucose decreased the number of CD11b^+^ cells. A possible explanation is that high glucose might decrease the proliferation of CD11b^+^ cells due to cell cycle arrest [38]. Nevertheless, there was an increase in ED1 levels in cells exposed to high glucose, indicating microglia activation. In fact, this possibility was supported by the increase in IL-1*β* levels when cells were exposed to high glucose, since activated microglia are prone to release increased levels of cytokines.

Similar to what was observed for microglial cells, IL-1*β* increased the number of GFAP^+^ cells, as well as the number of GFAP^+^ cells stained with Ki-67, indicating that glial cells are also proliferating. After injury, Müller cells become activated and undergo reactive gliosis, which is characterized by increased cell proliferation and gene expression alterations [39]. However, when retinal cells were exposed to high glucose, there was a decrease in GFAP levels and a decrease in the number of GFAP^+^ cells, indicating that elevated glucose per se has an inhibitory effect on glial cell proliferation, despite upregulating the levels of IL-1*β* in these cultures. The reduction in glial cell proliferation is not due to glial cell death [40]. These observations suggest that the effect of IL-1*β* on glial cell proliferation is clearly dependent on its concentration in the cell culture media. With relatively low levels of IL-1*β*, this cytokine is not able to induce glial cell proliferation.

Continuous stimulation with stress signals can lead to chronic microglia overstimulation with a consequent loss of its autoregulatory mechanisms, amplifying inflammation [41] which may consequently be harmful for neurons. When retinal cultures were exposed to IL-1*β* for 24 h, we did not detect any changes in the levels of TUJ-1 (data not shown), a neuronal marker. Nevertheless, we cannot exclude the possibility that for longer periods of exposure to IL-1*β*, microglia cells may have detrimental effects in these cultures changing from a protective to a proinflammatory modus [42, 43].

It was previously demonstrated that exposure to high glucose increases TNF expression [37]. The blockade of TNF receptor 1, which is expressed in retinal neurons, prevents high glucose-induced cell death. Additionally, it was demonstrated that the secretion of TNF and monocyte chemotactic protein-1 by rat cortical microglia, triggered by exposure to high glucose, is mediated by reactive oxygen species production and NF-*κ*B pathway activation, which may be mechanisms underlying neuronal injury and the pathogenesis of diabetic encephalopathy [44]. Moreover, IL-1RI-deficient mice are protected from diabetes-induced retinal pathology [45], indicating that IL-1*β* signalling may play a key role in the development of diabetic retinopathy. Taking into account that IL-1RI is a crucial locus of control of IL-1*β* activity, blocking IL-1RI activity should be considered as a possible therapeutic strategy for the treatment of diabetic retinopathy. In the retina, activated microglia and Müller cell responses are described not to be independent but involve bidirectional feedback signals that help initiate and propagate a coordinated adaptive response [46]. Therefore, in this study, changes in cell proliferation and expression of cell-specific markers suggest that there are adaptive responses that may help to limit neuronal cell death by directing and amplifying inflammatory processes in order to restore and maintain homeostasis in neuronal cell cultures.

## 5. Conclusions

In summary, our findings show that high glucose increases IL-1*β* production in retinal cell cultures. Moreover, we found that high glucose and IL-1*β* differently affect microglial and macroglial cells. High glucose decreased microglial and macroglial proliferation, whereas IL-1*β* increased their proliferation. These apparently opposing effects might be related with the levels of IL-1*β*. When cells are exposed to high glucose, the levels of IL-1*β* reached are significantly lower comparing to the condition when cells are exposed to IL-1*β* only. Other possible explanations might be the different exposure duration to IL-1*β* (7 days versus 24 h exposure) or the effect of high glucose on cell cycle arrest which may superimpose the potential proliferative effect of IL-1*β*.

Since overactivation of microglial cells may have deleterious effects in the retina, limiting IL-1*β*-mediated inflammatory processes could be a mechanism to prevent the progression of diabetic retinopathy.

## Figures and Tables

**Table 1:** List of primary antibodies.

Antibody	Western blot dilution	Immunocytochemistry dilution	Company
Mouse anti-TUJ-1	1 : 1000	1 : 1000	Covance
Mouse anti-GFAP	1 : 5000	1 : 500	Sigma
Mouse anti-CD11b	—	1 : 100	Serotec
Rabbit anti-Iba-1	—	1 : 200	Wako
Mouse anti-ED-1	1 : 1000	1 : 250	Serotec
Goat anti-IL-1*β*	—	1 : 50	RD
Mouse anti-IL-1RI	1 : 500	1 : 100	RD
Mouse anti-PCNA	1 : 500	—	Santa Cruz
Mouse anti-*β*-actin	1 : 5000	—	Sigma
Rabbit anti-Ki-67	—	1 : 100	Abcam
